# Routine versus needs-based MRI in patients with prolonged low back pain: a comparison of duration of treatment, number of clinical contacts and referrals to surgery

**DOI:** 10.1186/1746-1340-18-19

**Published:** 2010-07-09

**Authors:** Rikke K Jensen, Manniche Claus, Charlotte Leboeuf-Yde

**Affiliations:** 1 Research Department, Spine Centre of Southern Denmark, Østre Hougvej 55, 5500 Middelfart, Denmark; 2 Institute of Regional Health Services Research, University of Southern Denmark, Winsløwparken 19.3, 5000 Odense, Denmark

## Abstract

**Background:**

The routine use of radiology is normally discouraged in patients with low back pain (LBP). Magnetic Resonance Imaging (MRI) provides clinicians and patients with detailed knowledge of spinal structures and has no known physical side effects. It is possible that insight into the pathological changes in LBP patients could affect patient management. However, to our knowledge, this has never been tested. Until June 2006, all patients at our specialised out-patient public clinic were referred for MRI on the basis of clinical indications, economic constraints, and availability of MRI (the "needs-based MRI" group). As a new approach, we now refer all patients who meet certain criteria for routine up-front MRI before the clinical examination (the "routine MRI" group).

**Objectives:**

The aims of this study were to investigate if these two MRI approaches resulted in differences in: (1) duration of treatment, (2) number of contacts with clinicians, and (3) referral for surgery.

**Design:**

Comparison of two retrospective clinical cohorts.

**Method:**

Files were retrieved from consecutive patients in both groups. Criteria for referral were: (1) LBP or leg pain of at least 3 on an 11-point Numeric Rating Scale, (2) duration of present symptoms from 2 to12 months and (3) age above 18 years. A comparison was made between the "needs-based MRI" and "routine MRI" groups on the outcomes of duration of treatment and use of resources.

**Results:**

In all, 169 "needs-based MRI" and 208 "routine MRI" patient files were identified. The two groups were similar in age, sex, and severity of LBP. However, the median duration of treatment for the "needs-based MRI" group was 160 versus 115 days in the "routine MRI" group (p = 0.0001). The median number of contacts with clinicians for the "needs-based MRI" group was 4 versus 3 for the "routine MRI" group (p = 0.003). There was no difference between the two approaches in frequency of referral for back surgery (p = 0.81). When the direct clinical costs were compared, the "routine MRI" group was less costly but only by €11.

**Conclusion:**

In our clinic, the management strategy of routinely performing an up-front MRI at the start of treatment did reduce the duration of treatment and number of contacts with clinicians, and did not increase the rate of referral for back surgery. Also, the direct costs were not increased.

## Background

Immediate routine use of imaging in patients with low back pain (LBP) is currently discouraged by some experts in this area [1]. The reasons for this are that only few cases of serious pathology are found in the clinical population [2,3], little is known about the clinical relevance of other spinal pathological or degenerative findings [4], [5], [6], and access to these images seems to have little or no influence on treatment effect [7].

Magnetic Resonance Imaging (MRI) is increasingly replacing other imaging modalities in the diagnosis of LBP but the routine use of "up-front" MRI is not recommended [1]. An up-front MRI is an MRI which patients receive on a routine basis prior to the clinical examination.

From the patients' perspective, knowledge of various anomalies - many of which are normal degenerative findings - are, by some, thought to induce anxiety and dependence on health care services in those who are ill informed, which in turn could cause ill-advised medical interventions [8]. Others suggest that early use of MRI has a reassuring effect [7,9].

From a societal perspective, the cost of an MRI examination is high. Also, detailed visualisation of various abnormalities, such as a disc protrusion, could result in overzealous referral for surgery [7,10]. This could have both adverse economic consequences (because of the high cost of surgery) and negative personal consequences (because of the higher risk of serious side effects with surgery as compared with conservative treatment).

An additional perspective, however, is the growing trend for patients to distrust or disregard expert advice [11,12] as many clinicians have observed. Also, the medical profession is losing its traditional hold on the role of gate-keeper with full control over the management of the entire clinical course [13], [14], [15]. Today, many patients view health personnel in a given health field as just one of many sources of information and providers of services [16]. Therefore, if one health practitioner refuses to refer a patient for advanced imaging, the patient might continue his/her search for full information until an MRI has been obtained. This is possible because many patients have private insurance or may even pay themselves, and if the public system is uncooperative, there are private clinics that may be less restrictive in their criteria for proceeding with imaging.

On the one hand, this may have the positive effect of stopping the continued search for an MRI, but on the other hand, if the patient gets an unsuitable explanation, where findings are not explained in relation to the patient's specific spinal complaint, it may not result in an improvement of e.g. well-being, fear avoidance beliefs and avoidance of everyday activities [17,18].

As a consequence of this new development, which has accelerated in Denmark over the past few years, a new approach has been introduced into our specialised, out-patient public clinic. All patients with LBP referred to the clinic, who fulfil certain inclusion criteria, have since June 2006 received an up-front routine MRI examination on the first visit. This occurs before being examined by a clinician, rather than on a perceived needs basis.

The introduction of this new approach was based on the assumption that up-front access to an MRI report will have an anxiety-reducing effect when the patient learns that there is nothing seriously wrong. Also, if there is no effect of the treatment, consisting of exercise-based conservative therapy, the duration of treatment at the clinic does not have to be prolonged while waiting for the required MRI that might enlighten the clinician further. Having the anatomical facts at hand is thought to make it easier for both patient and clinician to accept the situation for what it is. This in turn is thought to effect the duration of treatment, reduce the risk of chronicity and sick-leave, and hence save society unnecessary costs. A quicker turn-over of patients will also have the benefit of reducing the waiting lists at this specialised clinic.

Nevertheless, to our knowledge, these potentially positive aspects of up-front routine MRI in patients with prolonged LBP have not been studied. For this reason, we made use of the standardised records available in the clinic, and performed a study that compared the present system with that previously used. We were able to retrieve information on, and compare the duration of, treatment, number of contacts with clinicians, and referral for surgery that occurred before and after the practice of routine MRI. However, we did not have access to information on any relevant psychosocial data, making it impossible to study patients' personal reactions and indirect costs. Nevertheless, the direct costs relating to the MRI and the subsequent visits to the clinic could be identified. A crude analysis was therefore performed comparing these costs in the two groups.

## Method

### Design

The study involved a comparison of two retrospective clinical cohorts.

### Flow of study

A comparison was made between two patient cohorts that differed only on the method by which MRI was prescribed. During the period when the study was carried out, no other procedures were changed in the clinic. All had attended the same specialised outpatient spine clinic in Denmark (Spine Centre of Southern Denmark, Ringe) after referral from the primary care sector. Criteria for referral were: (1) back problems with or without radiculopathy, (2) duration of the actual episode being a maximum of two years, and (3) appropriate treatment that was unsuccessful in the primary care setting.

A hand search was done for the two groups, "routine MRI" and "needs-based MRI", in order to collect information that made it possible to ascertain whether the two methods of MRI prescription had an apparent effect on the duration of treatment and the use of resources.

### Study participants

#### "Routine MRI" group

From June 2006, MRI was performed on all patients meeting the following criteria: (1) LBP or leg pain of at least 3 on an 11-point Numeric Rating Scale, (2) duration of present symptoms from 2 to 12 months, and (3) age above 18 years. Information was obtained on all patients who had attended the clinic from June 2006 till the time of the study (February 2007), including both baseline and outcome data.

#### "Needs-based MRI" group

Up until June 2006, patients at the clinic received an MRI purely on the basis of clinical indications as determined during the course of the examination and treatment. A computerised list of all patients who attended the clinic between January and December 2005 was obtained. On the basis of the date of birth, the patient files were manually retrieved in order to select those who met the same criteria as those in the "routine MRI" group. The search was stopped at an arbitrary number anticipated to correspond to the approximate number of participants in the "routine MRI" group. The same information was collected as for the "routine MRI" group, together with information about referral for MRI.

### Variables of interest

The following baseline variables were obtained from the standard baseline questionnaire which was included in the patient file: sex, age, severity of low back pain (11-point Numeric Rating Scale), leg pain (11-point Numeric Rating Scale), disability (LBP Rating Scale) [19], and duration of symptoms (months in pain).

Three main outcome variables were obtained from the computerised booking system after the end of treatment. These were: (1) Duration of time until referral back to the primary sector or other health care provider (date of referral back minus date of first visit), (2) Number of visits to the clinic (counted from the booking system), (3) Referral for spine surgery (based on a specific code in the booking system) and (4) If an MRI was performed (verified from the date of MRI in the booking system).

The direct costs of an MRI and a visit to the clinic were estimated from the National Health Service of Denmark by DRG rates (Diagnosed Related Grouping), using rates from 2007 [20].

### Analysis of data

Initially, the baseline variables for the "needs-based MRI" and "routine MRI" were compared to see if they resembled each other. The two groups were then compared on the outcome variables mentioned above. As most variables were non-normally distributed, non-parametric inferential statistics were used (Wilcoxon rank sum test).

As it is our experience that patients with dominating leg pain often have a longer course of treatment and a worse prognosis than those with mainly back pain, those with leg pain were initially analyzed separately on the three outcome variables. However, as no differences were found (p = 0.08 to 0.97), they were subsequently analyzed together.

We used the rates from the National Health Service of Denmark [20] to calculate the total costs on MRIs and visits for each group. The cost per patient was estimated by dividing the total cost by the number of patients in each group. Danish kroner were converted into Euros with the current exchange rate of DKK 7.44 to EUR 1.

## Results

### Description of the two cohorts

In all, 169 "needs-based MRI" and 208 "routine MRI" patient files were identified. Forty-three percent of the patients in the "needs-based MRI" group had an MRI compared with everybody in the "routine MRI". The two groups were similar in relation to age, sex, severity of back pain and leg pain, and functional disability (Table 1). The median age for both groups was 48 years and there was almost an even distribution of men and women. The median for leg pain and back pain was 5 on an 11-point Numeric Rating Scale and the functional disability score was around 50%. However, there was a difference in duration of symptoms, with a total range from 2 to12 months; the median estimate for the "routine MRI" group was 5 months, but only 4 months for the "needs-based MRI" group. The actual results are shown in Table 1.

**Table 1:** Description of the two cohorts and outcome data.

Description of the two cohorts	Needs-based MRI (169)	%	Routine MRI (208)	%	p-value
Referred to MRI	72	43	208	100	
**Age (years)**					
Median	48	-	48	-	0.88*
Quartiles 25-75	39-56	-	37-58	-	-
**Sex**					
Men	83	49	110	53	-
Women	86	51	98	47	-
**Back pain (0-10)**					
Median	5	-	5	-	0.14*
Quartiles 25-75	4-7	-	4-7	-	-
**Leg pain (0-10)**					
Median	5	-	5	-	0.95*
Quartiles 25-75	2-7	-	2-7	-	-
**Disability (%)**					
Median	-	50	-	54.5	0.16*
Quartiles 25-75	-	37-68	-	42-69	-
**Duration of symptoms (months)**					
Median	4	-	5	-	0.04*
Quartiles 25-75	3-6	-	3-7	-	-

**Outcome**	**Needs-based MRI**	**%**	**Routine MRI**	**%**	**p-value**

**Referred to surgery**	15	9	17	8	0.81*
**Duration at clinic (days)**					
Median	160	-	115	-	0.0001*
Quartiles 25-75	106-122	-	75-161	-	-
**Visits at clinic (number)**					
Median	4	-	3	-	0.003*
Quartiles 25-75	2-7	-	2-5	-	-

* Wilcoxon rank sum test

### Outcome

The median duration of treatment for the "needs-based MRI" group was 160 versus 115 days in the "routine MRI" groups (p = 0.0001). The median number of visits to the clinic for the "needs-based MRI" group was 4 versus 3 for the "routine MRI" group (p = 0.003). There was no difference between the two groups in relation to referral for back surgery (p = 0.81).

### Costs

For a patient in the "needs-based" group, the direct cost for MRI and other clinical consultations was €968 compared with €957 for a patient in the "routine MRI" group. For details see Table 2.

**Table 2:** Calculations of direct costs.

	Price	Needs-based group (n = 169)		Routine MRI (n = 208)	
MRI	€332.43	72	€23934.96	208	€69145.44
Visit	€176.70	790	€139593.00	735	€129874.50
**Costs**					
Total per group			€163527.96		€199019.94
Total per patient			€967.62		€956.83

## Discussion

When referral for MRI occurred within the "needs-based MRI" system, that is, when it was based on clinical reasoning and experience, the duration of treatment was longer with more visits to the clinic. However, when using the new approach, where all patients were routinely referred for MRI, the duration of treatment was reduced, as was the number of visits. At the same time, there was no increase in the rate of referral for surgery. For the "needs-based MRI" group, the direct costs of clinical consultations were higher but the cost for MRIs were lower than in the "routine MRI" group. Overall, the per patient total costs were similar between the groups.

In a systematic review [1] that compared the effect of early routine lumbar imaging with usual clinical care without up-front imaging, only two studies investigated MRI [9,18]. Those studies involved patients with LBP where there was no indication of serious underlying conditions. The results were mixed, based on patient-reported information, for example: pain, disability, quality of life and mental health.

Our study used a different approach. We investigated this issue from a more administrative/logistics perspective, and from this point of view, our results were unambiguously in favour of the routine use of MRI for this type of patient. To our knowledge, this is the first time the issue about the routine use of up-front MRI has been investigated from a non-clinical perspective.

When the direct clinical costs were compared, the "routine MRI" group was less costly but only by €11. However, costs would possibly differ from setting to setting, depending on the type of governmental payment structure, type of treatment and structure of treatment program. Our results are based on a crude calculation of the direct costs, which were the only economic data available. A full economic evaluation would be necessary to make definitive conclusions about the cost-effectiveness of this new MRI approach.

Our results are based on a retrospective study with a historical control group and the differences between the two groups could be influenced by time-related bias, for example: different clinicians at different time points, availability of MRI or change in management in the clinic, or it could be due to other procedural differences at the two time points. To test this more precisely, would require a randomised controlled trial.

However, even if a randomised controlled trial showed convincing results against up-front MRI, the implementation of such findings may be difficult in a country such as Denmark, where patients are influential on clinical decision-making. If a patient is not satisfied with the decision of the practitioner, the risk of the patient's "doctor shopping" is high.

An argument against the routine use of MRI is that MRI is expensive [21]. Nevertheless the costs of an MRI scan might be extremely different in various countries and over time. In Denmark, the costs are regulated by the authorities in the health sector and heavily influenced by the insurance companies on the free market. The costs of a prolonged duration of treatment and multiple visits to clinicians might be less if an up-front MRI scan resulted in an earlier closure of the clinical course and precluded the expenses associated with "doctor-shopping". The results of this study suggest that there is a need for further studies of both the cost-effectiveness and patient outcomes that result from different approaches to MRI use in managing low back pain.

## Conclusion

In a health care system where patients can disregard a clinical decision not to have an MRI, as is the case in Denmark, the use of up-front routine MRI appears to be an effective method to optimise patient flow through a secondary care back pain centre. Further research should investigate whether up-front MRI leads to improved patient outcomes and is cost-effective in other clinical settings.

## Competing interests

The authors declare that they have no competing interests.

## Authors' contributions

RKJ participated in conception and design, carried out the data collection and the analysis, and main parts of the manuscript. CM participated in the design and coordination of the study and helped to draft the manuscript. CLY participated in the conception and made substantial contributions to the manuscript. All authors have read and approved the final manuscript.
